# Antigenicity and Immunogenicity of *Plasmodium vivax* Merozoite Surface Protein-3

**DOI:** 10.1371/journal.pone.0056061

**Published:** 2013-02-14

**Authors:** Amanda R. Bitencourt, Elaine C. Vicentin, Maria C. Jimenez, Ricardo Ricci, Juliana A. Leite, Fabio T. Costa, Luis C. Ferreira, Bruce Russell, François Nosten, Laurent Rénia, Mary R. Galinski, John W. Barnwell, Mauricio M. Rodrigues, Irene S. Soares

**Affiliations:** 1 Departamento de Análises Clínicas e Toxicológicas, Faculdade de Ciências Farmacêuticas, Universidade de São Paulo, São Paulo, Brazil; 2 Departamento de Genética, Evolução e Bioagentes, Instituto de Biologia, Universidade Estadual de Campinas, Campinas, Brazil; 3 Departamento de Microbiologia, Instituto de Ciências Biomédicas, Universidade de São Paulo, São Paulo, Brazil; 4 Department of Microbiology, Yong Loo Lin School of Medicine, National University of Singapore, Singapore; 5 Singapore Immunology Network, Biopolis, Agency for Science Technology and Research, Singapore, Singapore; 6 Centre for Vaccinology and Tropical Medicine, Churchill Hospital, Oxford, United Kingdom; 7 Mahidol–Oxford University Tropical Medicine Research Programme, Shoklo Malaria Research Unit, Mae Sot, Thailand; 8 Emory Vaccine Center and Yerkes National Primate Research Center, Emory University, Atlanta, Georgia, United States of America; 9 Department of Medicine, Division of Infectious Diseases, Emory University, Atlanta, Georgia, United States of America; 10 Malaria Branch, Division of Parasitic Diseases, Centers for Disease Control and Prevention, Chamblee, Georgia, United States of America; 11 CTCMOL, Departamento de Microbiologia, Imunologia e Parasitologia, Universidade Federal de São Paulo-Escola Paulista de Medicina, São Paulo, Brazil; Centro de Pesquisa Rene Rachou/Fundação Oswaldo Cruz (Fiocruz-Minas), Brazil

## Abstract

A recent clinical trial in African children demonstrated the potential utility of merozoite surface protein (MSP)-3 as a vaccine against *Plasmodium falciparum* malaria. The present study evaluated the use of *Plasmodium vivax* MSP-3 (PvMSP-3) as a target antigen in vaccine formulations against malaria caused by *P. vivax*. Recombinant proteins representing MSP-3α and MSP-3β of *P. vivax* were expressed as soluble histidine-tagged bacterial fusions. Antigenicity during natural infection was evaluated by detecting specific antibodies using sera from individuals living in endemic areas of Brazil. A large proportion of infected individuals presented IgG antibodies to PvMSP-3α (68.2%) and at least 1 recombinant protein representing PvMSP-3β (79.1%). In spite of the large responder frequency, reactivity to both antigens was significantly lower than was observed for the immunodominant epitope present on the 19-kDa C-terminal region of PvMSP-1. Immunogenicity of the recombinant proteins was studied in mice in the absence or presence of different adjuvant formulations. PvMSP-3β, but not PvMSP-3α, induced a TLR4-independent humoral immune response in the absence of any adjuvant formulation. The immunogenicity of the recombinant antigens were also tested in formulations containing different adjuvants (Alum, *Salmonella enterica* flagellin, CpG, Quil A,TiterMax® and incomplete Freunds adjuvant) and combinations of two adjuvants (Alum plus flagellin, and CpG plus flagellin). Recombinant PvMSP-3α and PvMSP-3β elicited higher antibody titers capable of recognizing *P. vivax-*infected erythrocytes harvested from malaria patients. Our results confirm that *P. vivax* MSP-3 antigens are immunogenic during natural infection, and the corresponding recombinant proteins may be useful in elucidating their vaccine potential.

## Introduction

Recent studies have made important advances toward the development of a vaccine against human malaria caused by *Plasmodium falciparum*. Clinical trials performed in African endemic areas demonstrated 3 distinct antigens have a significant, albeit partial, effect in retarding clinical malaria acquisition in vaccinated children. These antigens are the circumsporozoite protein (CSP), apical membrane antigen-1 (AMA-1), and merozoite surface protein (MSP)-3 [1]–[3]. These results confirm the feasibility of an effective malaria vaccine.

In contrast to *P. falciparum* malaria, vaccine development against *P. vivax* malaria lags far behind. Few phase I clinical trials have been performed and phase II trials have yet to be initiated [4]–[6]. This is a significant hurdle for malaria eradication, as a vaccine against *P. vivax* is an essential step toward this objective [7].

To reduce the gap in the development of a vaccine against *P. vivax* malaria, we and others have worked for the past 15 years, characterizing naturally acquired immune responses to pre-erythrocytic and blood-stage recombinant antigens in individuals from endemic areas of South America [8]–[20]. A number of pre-clinical studies in mice and non-human primates were performed using these recombinant antigens. These pre-clinical studies used recombinant or synthetic antigens based on the CSP, MSP-1, AMA-1, and Duffy-binding protein [21]–[27].

PfMSP-3.1 provided protective immunity in African children vaccinated against *P. falciparum* infection [3], providing important evidence that a comparable antigen from *P. vivax* may also be a viable candidate for the development of a vaccine against *vivax* malaria. In *P. vivax*, MSP-3 (PvMSP-3) comprises a family of proteins characterized by a putative signal peptide, a central alanine-rich domain, and the lack of a C-terminal transmembrane domain or GPI anchor motif [28]–[30]. PvMSP-3α and PvMSP-3β were initially predicted to form α-helical secondary and coiled-coil tertiary structures with heptad repeats [28], [29]. We recently demonstrated that recombinant proteins based on predicted coiled-coil domains of PvMSP-3α form oligomeric and elongated molecules [31], suggesting this protein may mediate interactions with host proteins or other merozoite surface proteins.

Based on the promising results of vaccination with *P. falciparum* MSP-3.1 (the one member of the PfMSP3 family that has a central domain of predicted coiled-coil structure [32]), this study was designed to evaluate the antigenicity of four prokaryotic recombinant proteins representing PvMSP-3α or PvMSP-3β of *P. vivax* in humans and mice.

## Materials and Methods

### Ethics Statement

Blood samples were obtained for research use with the written informed consent of all study participants enrolled in a protocol approved by the Ethics Committee of the Faculty of Pharmaceutical Sciences of University of São Paulo, Brazil (CEP No. 22/2001), the Ethics Committee of the Faculty of Tropical Medicine, Mahidol University, Thailand (MUTM 2010-006-01), and the University of Oxford, Centre for Clinical Vaccinology and Tropical Medicine, United Kingdom (OXTREC 027-025). This study was performed in strict accordance with the recommendations in the Guide for the Care and Use of Laboratory Animals of the Brazilian National Council of Animal Experimentation (http://www.cobea.org.br/). The protocol was approved by the Committee on the Ethics of Animal Experiments of the Faculty of Pharmaceutical Sciences of University of São Paulo, Brazil (CEEA No. 112/2006).

### Subjects

Serum samples were collected from 220 individuals with patent *P. vivax* malaria in five different localities of the Amazon Region and described in detail elsewhere [9], [11]. These samples were tested for the presence of IgG antibodies against the C-terminal region of MSP-1 (PvMSP1_19_), apical membrane antigen-1 (AMA-1), and the Duffy binding protein (PvRII) [11], [13], [16]. A second group was composed of 26 healthy adult volunteers selected from blood donors in the city of São Paulo, State of São Paulo, southeastern Brazil (control group).

### Recombinant Proteins

The recombinant proteins presented in Table 1 were expressed in *Escherichia coli* as described elsewhere [21], [31]. Briefly, *E. coli* BL21-DE3 (Novagen) containing the recombinant plasmids pHISa-MSP-3α, pHISa-MSP-3β (FP-1), pHISb-MSP-3β (FP-2), pET14b-MSP-3β (FP-3), and pET14b-MSP1_19_ were cultivated in 1 L of LB-ampicillin (100 µg/mL) at 37°C shaken culture to OD_600_ 0.6–0.8. Recombinant protein expression was induced by 3 h incubation with 0.1 mM isopropyl-β-d thiogalactopyranoside (IPTG, Life Technologies). The bacterial supernatant was obtained by centrifugation at 24,000 *g* for 60 min at 4°C and recombinant proteins were purified by affinity chromatography on Ni^2+^-NTA Agarose (Qiagen) following by AKTA Prime using anionic-columns (GE Healthcare). Fractions were analyzed by SDS-PAGE and stained with Coomassie blue. Fractions containing recombinant proteins with a high degree of purity were pooled and extensively dialyzed against PBS. The protein concentration was determined spectrophotometrically at 280 nm. All batches of recombinant proteins were tested by circular dichroism spectroscopy, as described previously [31].

**Table 1:** Recombinant proteins used in the immunological studies.

*Protein*	*Fragment Name*	*Sequence*	*Expression vector*	*Apparent molecular weights*
*PvMSP-3α*	*FP-1*	*359–798*	*pHISa*	*87 kDa*
PvMSP-3β	FP-1	*35–375*	*pHISa*	*60 kDa*
PvMSP-3β	FP-2	*385–654*	*pHISb*	*57 kDa*
PvMSP-3β	FP-3	*35–654*	*pET14b*	*104 kDa*
PvMSP-1	MSP1_19_	*1616–1704*	*pET14b*	*18 kDa*

*FP = Fusion Protein with His-tag.*

### Adjuvants and Mouse Immunization

Initially, 4 groups of C57BL/6 (H-2^b^) and C57BL/6 TLR4 knockout (TLR4 KO, non-responsive to LPS) mice were immunized subcutaneously (s.c.) with 10 µg of each recombinant PvMSP-3 in the absence of adjuvants. Animals of 6–8 week-old were purchased from Federal University of São Paulo, Brazil. A volume of 50 µL was injected into each footpad. After 15 and 30 days, each animal received a booster injection of 10 µg of the same protein injected s.c. at the base of the tail.

PvMSP-3α and PvMSP-3β (FP-3) were selected for immunization of BALB/c (H-2^d^) mice. The animals were purchased from University of São Paulo, Brazil. The immunization schedule was the same as for C57BL/6 mice, except that the animals were immunized in the presence of 6 adjuvants. In order, the antigens included 25 µg of Imject® Alum (Pierce), 2.5 µg of FliC flagellin of *Salmonella enterica* Typhimurium, 10 µg of CpG-ODN 1826 (TCCATGACGTTCCTGACGTT) (Prodimol Biotecnologia), 25 µg of Quil A (Superfos Biosector), or an equal volume of TiterMax Gold (Sigma) or Incomplete Freund’s Adjuvant (IFA). PvMSP-3β (FP-3) was also co-administered in CPG ODN 1826 plus Alum or FliC. These adjuvants were administered at the doses used for immunization with single adjuvants. Controls received only PBS emulsified in adjuvant. Serum samples were collected for analysis 14 days after each dose and stored at -20°C.

### Immunological Assays

#### ELISA detection of human IgG antibodies

Human IgG antibodies against PvMSP-3α, PvMSP-3β (FP-1, FP-2, and FP-3), and PvMSP1_19_ of *P. vivax* were detected by ELISA [11]. ELISA plates were coated with 200 ng/well of each recombinant protein. Fifty microliters of each solution were added to each well of a 96-well plate (High binding, Costar). After overnight incubation at room temperature (r.t.), the plates were washed with PBS-Tween (0.05%, v/v) and blocked with PBS-milk (PBS, pH 7.4, containing 5% nonfat dry milk) for 2 h at 37°C. Serum samples were diluted 1∶100 in the same solution and 50 µL of each sample was added to duplicate wells. After incubation for 2 h at r.t. and washes with PBS-Tween, 50 µL of a solution containing peroxidase-conjugated goat anti-human IgG (Fc-specific) diluted 1∶5.000 (Sigma) was added to each well. The enzymatic reaction was developed by the addition of 1 mg/mL *o*-p-phenylenediamine (Sigma) diluted in phosphate-citrate buffer, pH 5.0, containing 0.03% (v/v) hydrogen peroxide, and stopped by the addition of 50 µL of 4 N H_2_SO_4_. Plates were read at 492 nm (OD_492_) with an ELISA reader (Awareness Technology, mod. Stat Fax 2100, EUA). Cutoff points were set at 3 standard deviations above the mean OD_492_ of sera from 26 individuals, unexposed to malaria, from the city of São Paulo. The results are expressed as index of reactivity (IR).

#### ELISA detection of mouse antibodies

Antibodies to PvMSP-3 in mouse sera were detected by ELISA on days 14, 29, and 44 as described previously [33]. ELISA plates (High binding, Costar) were coated with 200 ng/well of the homologous recombinant protein. Each solution (50 µL) was added to each well of a 96-well plate. After overnight incubation at r.t., the plates were washed with PBS-Tween and blocked with PBS-milk-BSA (PBS, pH 7.4, containing 5% nonfat dry milk, 2.5% BSA) for 2 h at 37°C. Mouse sera were tested in serial dilutions starting at 1∶100; a final volume of 50 µL of sample was added to duplicate wells, following incubation for 1 h at r.t. After washes with PBS-Tween, 50 µL of a solution containing secondary antibody conjugated to peroxidase (goat anti-mouse IgG, KPL) diluted 1∶3.000 was added. The enzymatic reaction was developed as described for ELISA detection of human IgG antibodies. Detection of IgG subclass responses was performed as described above, except the secondary antibody was specific to mouse IgG1, IgG2a, IgG2b or IgG3 (Southern Technologies) diluted 1∶8.000. The specific anti-PvMSP-3 titers were determined as the highest dilution yielding an OD_492_ greater than 0.1. The results are expressed as means of IgG titers (Log_10_) ± SEM.

#### *P. vivax* slide preparation and immunofluorescence assays

The thin smears used for the IFA were prepared from *ex vivo* matured and 45% percoll concentrated schizonts [34] that were diluted 1∶4 with uninfected RBCs to provide enough volume to make a number of duplicate slides. Clinical isolates of *P. vivax*-infected blood from malaria patients were collected at Shoklo Malaria Research Unit (Thailand) with written informed consent. Pooled sera from mice immunized 3 times with the recombinant PvMSP-3α and PvMSP-3β (FP-3) proteins emulsified in Freund’s Adjuvant (1∶100) were applied to the smear and incubated for 1 h before incubation with secondary anti-mouse IgG antibody conjugated to Alexa Fluor 568 (Invitrogen) or DAPI (4′,6-diamidino-2-phenylindole, dihydrochloride, Invitrogen). The presence of native PvMSP-3 was visualized using a Nikon TS100 epifluorescence microscope.

### Statistical Analysis

Differences between the proportions of responder individuals were analyzed by the Chi-square test. Comparison of antibody level (IR) in independent samples was performed by One-way analysis of variance (ANOVA) and correlations were determined by the nonparametric Spearman correlation coefficient. One-way ANOVA was used to compare normally distributed log-transformed means for the different animal groups. Multiple comparisons were assessed by Tukey’s Test, with a P-value of <0.05 considered significant.

## Results

### Antigenicity of Recombinant PvMSP-3α or PvMSP-3β Proteins

**Figure 1:**
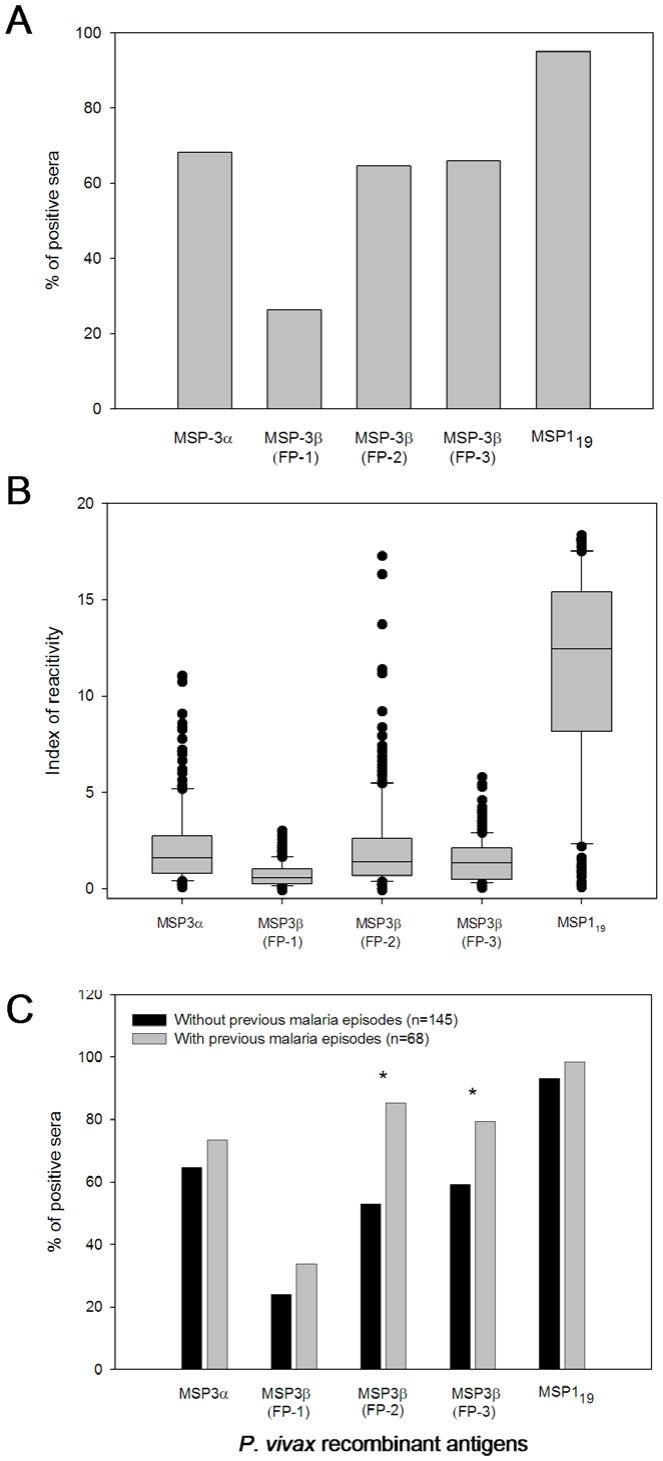
Human antibody response to recombinant PvMSP-3α, PvMSP-3β, and PvMSP1_19_ proteins during patent *P. vivax* infection. **A**) The bars express the percent response for each of the analyzed proteins. Sera from 220 individuals were analyzed for the presence of specific IgG antibodies by ELISA and tested at a 1∶100 dilution in duplicate. The cutoff proteins obtained from the PvMSP-3α, PvMSP-3β (FP-1), PvMSP-3β (FP-2), PvMSP-3β (FP-3), and PvMSP1_19_ were 0.179, 0.715, 0.195, 0.396, and 0.185, respectively. **B)** Comparison of individual IR IgG antibodies to MSPs in sera of individuals infected by *P. vivax*. The line indicates the limit of positivity (IR = 1). IR: Index of reactivity (mean absorbance of test serum/cutoff). **C)** Comparative analysis of the IgG antibody response against MSP proteins and the frequency of previous episodes of *vivax* malaria. We analyzed 213 serum samples from individuals who reported the number of previous episodes of malaria for the presence of specific IgG antibodies by ELISA. All sera were tested in duplicate at 1∶100 dilution. *: the percentage of responders with statistically significant correlation to the frequency of previous malaria episodes.

Initially, we compared the IgG antibody response of individuals during patent infection to the recombinant PvcMSP-3α and PvMSP-3β (FP-1, FP-2, and FP-3) Proteins. Details of each recombinant protein can be found in our previously published studies on the biochemistry of these proteins [31]. The frequency of responders to MSP-3α and at least one recombinant protein representing PvMSP-3β was 68.2% and 79.1%, respectively, indicating both proteins are immunogenic during infection with *P. vivax*. The frequency of individuals presenting IgG antibodies to each recombinant PvMSP-3β was 26.3% (FP-1), 64.5% (FP-2), and 65.9% (FP-3). The prevalence of antibodies against FP-1 was significantly lower than the prevalence of antibodies against FP-2 and FP-3 of PvMSP-3β (Chi-square test, *p*<0.001). Reactivity to FP-2 and FP-3 of PvMSP-3β did not statistically differ (*p>*0.05). We also compared the reactivity of each PvMSP-3 recombinant protein with an immunodominant epitope of *P. vivax*, contained within the PvMSP1_19_ protein [9], [11]. The responder frequency and reactivity index in malaria-infected individuals with PvMSP1_19_ were significantly higher when compared to the other recombinant proteins (*p*<0.001, Figure 1A and B, respectively).

After repeated exposure, differences in reactivity between PvMSP1_19_ and the recombinant PvMSP-3 proteins were maintained (*p*<0.0001 to PvMSP-3α and PvMSP-3β FP-1, *p*<0.01 to PvMSP-3β FP-2, and *p*<0.001 to PvMSP-3β FP-3, Figure 1C). The proportions of responders to FP-2 and FP-3 increased significantly after repeated *P. vivax* infection reaching more than 80% in multiply infected individuals, indicating most individuals may become responders based on their degree of exposure. Such a pattern was not observed in response to FP-1 (Figure 1C).

**Figure 2:**
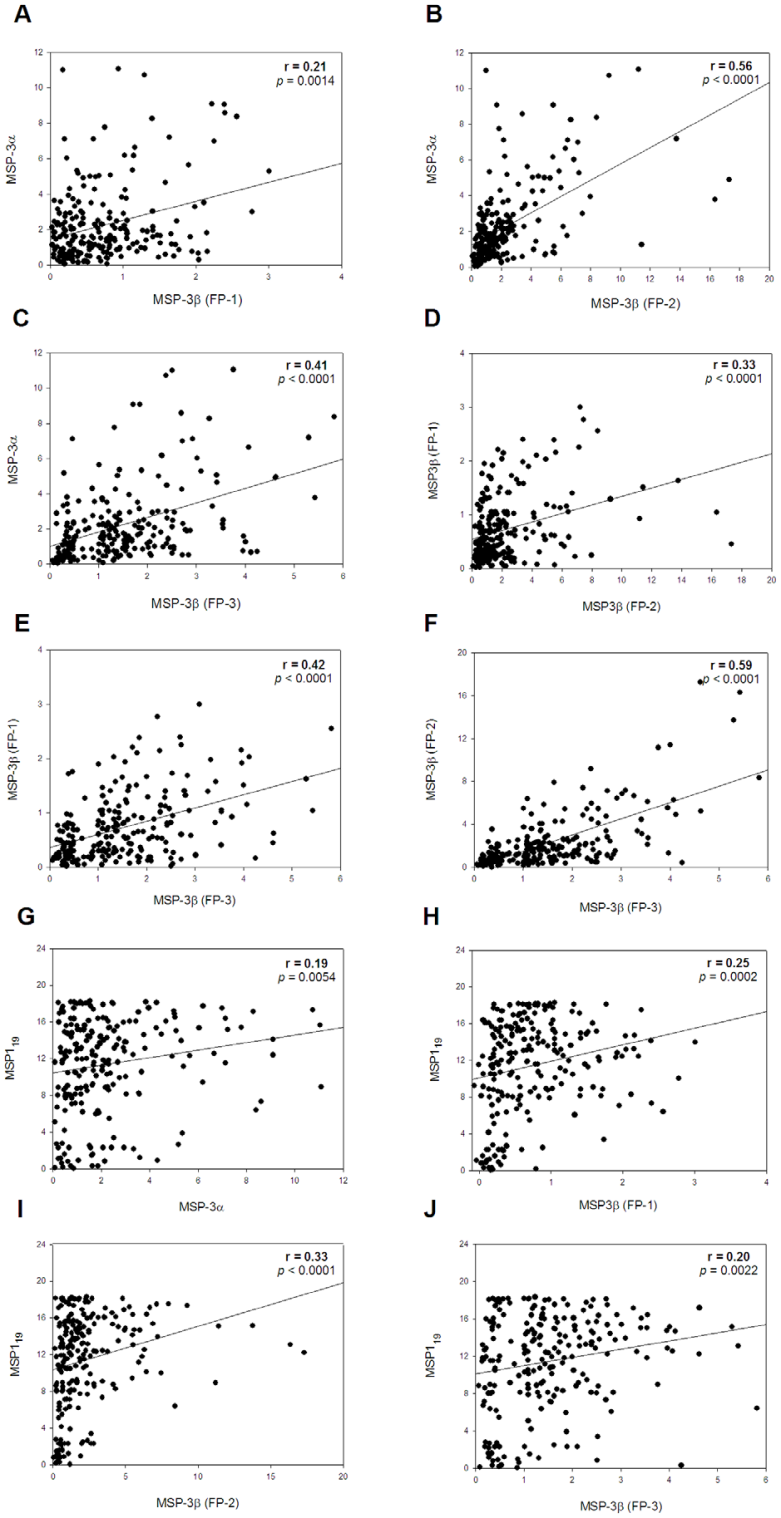
Reactivity against recombinant PvMSP-3 and PvMSP1_19_ proteins in 220 sera from individuals with patent *P. vivax* malaria infection. Each panel represents the reactivity index of serum samples against the indicated recombinant proteins. The serum samples were tested at a 1∶100 dilution, as described in Figure 1B. Symbols represent the IR IgG antibodies against recombinant MSP proteins in the sera of *P. vivax*-infected individuals. The values of the Spearman correlation coefficient (r) and *p* values are shown in each panel.

We also evaluated the correlation between antibody reactivities to paired recombinant proteins during patent infection. In all cases, significant correlations were observed. For example, there was a relatively high correlation between PvMSP-3α and PvMSP-3β FP-2 (r = 0.56, *p*<0.0001; Figure 2B), or PvMSP-3β FP-2 and PvMSP-3β FP-3 (r = 0.59, *p*<0.0001; Figure 2F), and a moderate correlation between PvMSP-3α and PvMSP-3β FP-3 (r = 0.41, *p*<0.0001; Figure 2C) or PvMSP-3β FP-1 and PvMSP-3β FP-2 (r = 0.33, *p*<0.0001; Figure 2D), or PvMSP-3β FP-1 and FP-3 (r = 0.42, *p*<0.0001; Figure 2E). Overall, the correlations between the antibody reactivities to PvMSP1_19_ and the other recombinant proteins (Figures 2G, 2H, 2I, and 2J) were weak.

### Immunogenicity of Recombinant PvMSP-3α and PvMSP-3β Proteins in Mice

**Figure 3:**
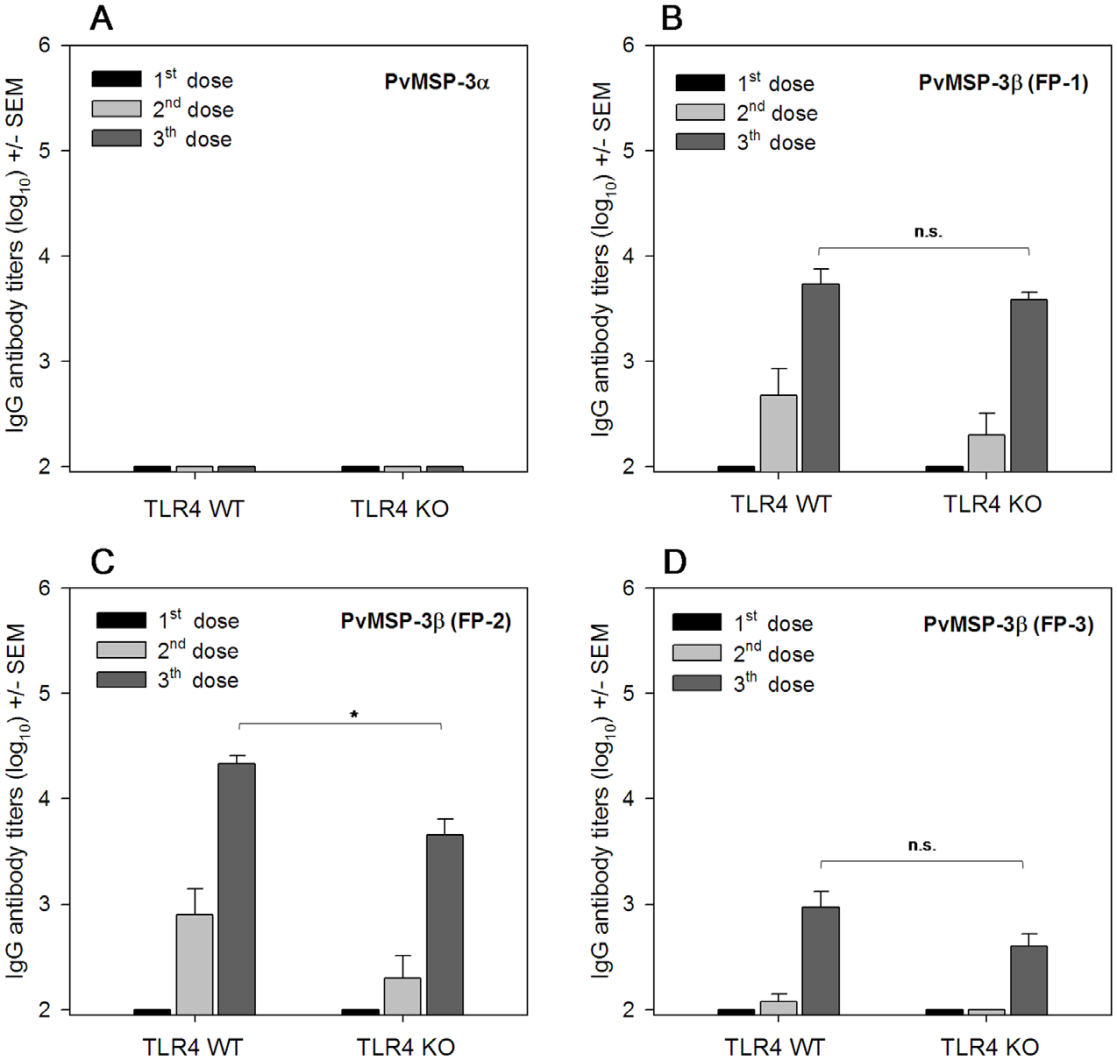
IgG antibody response in C57BL/6 wild-type (WT) and TLR4 KO mice after immunization with MSP-3 in the absence of adjuvant. Groups of 5 mice were immunized 3 times (s.c.) with 10 µg of PvMSP-3α, PvMSP-3β (FP-1), PvMSP-3β (FP-2), or PvMSP-3β (FP-3) and antibody titers to homologous PvMSP-3 were determined after each dose. Results are expressed as the means of antibody titers (log_10_) ± SEM and were compared by one-way ANOVA followed by Tukey’s test for multiple comparisons. After the third dose, non-significant differences between groups of immunized mice (C57BL/6 WT vs. TKR4 KO) are denoted on the graph as “n.s.” Significant difference between 2 groups of mice immunized with 3 doses of PvMSP-3β (FP-2) are denoted on the graph (*p<0.05).

The immunogenicity of recombinant proteins representing PvMSP-3α and PvMSP-3β was evaluated after immunization of C57BL/6 wild type (WT) mice. Animals immunized with PvMSP-3α failed to respond even after three immunizing doses (Figure 3A). In contrast, we detected specific antibodies in mice immunized 2 or 3 times with any of the PvMSP-3β proteins (Figures 3B, 3C, 3D). The antibody titers to PvMSP-3β FP-2 were higher than the titers to PvMSP-3β FP-1 or FP3 (*p*<0.01). In addition, the titers to PvMSP-3β FP-1 were higher than the titers to FP-3 (*p*<0.01, Figures 3B, 3C, and 3D).

Because bacterial recombinant proteins are often contaminated with LPS, we also compared the WT response to that of LPS-unresponsive TLR4 KO mice.

Similar titers were detected in WT and TLR4 KO mice following immunization with PvMSP-3β FP-1 and FP-3 (Figures 3B and 3D). In the case of PvMSP-3β FP-2, the titers of WT mice were significantly higher than in the TLR4 KO mice (*p*<0.05), suggesting a possible effect of LPS contamination in the protein preparation (Figure 3C ). Nevertheless, specific antibodies are still observed after PvMSP-3β FP-2 immunization.

### Adjuvant and Antibody Responses after Immunization with PvMSP-3α and PvMSP-3β (FP-3)

**Figure 4:**
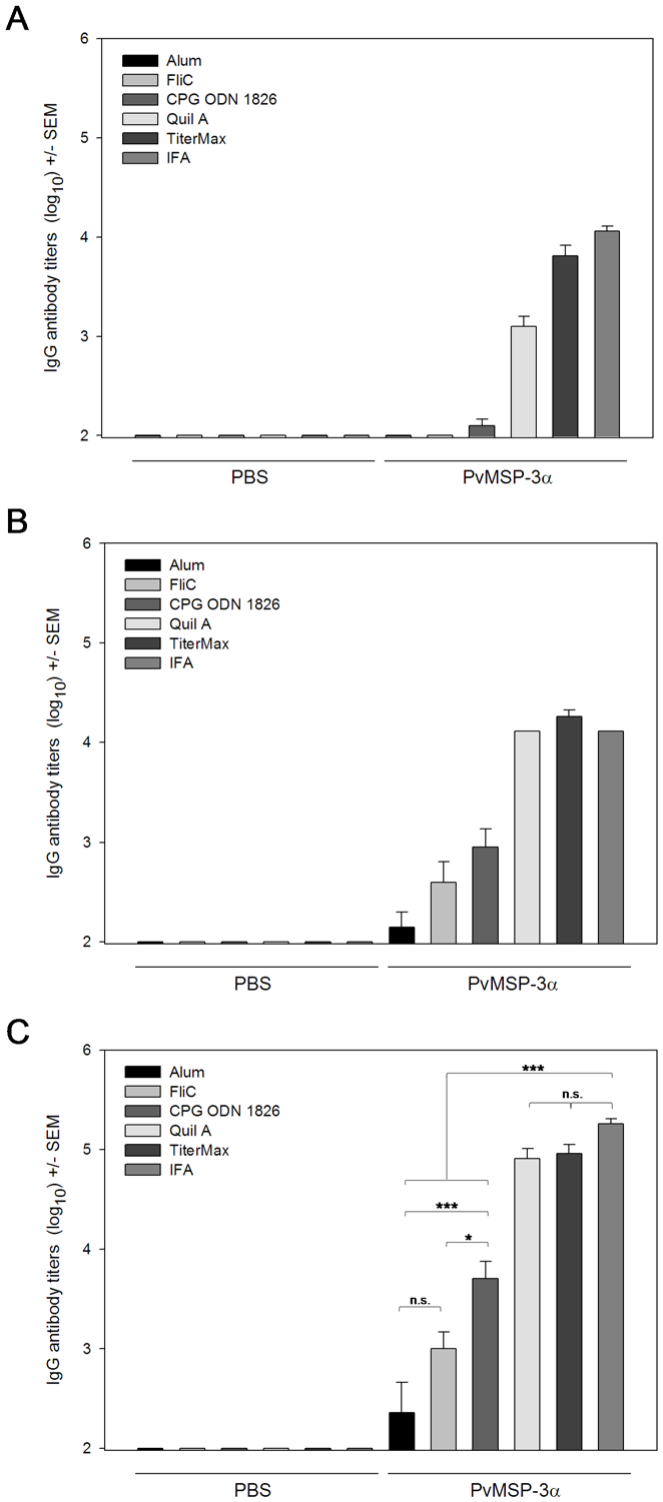
IgG anti-PvMSP-3α in mice immunized with various adjuvant formulations. Groups of 6 female BALB/c mice were immunized 3 times (s.c.) with 10 µg of protein in the following adjuvant formulations: Alum, FliC, CpG ODN 1826, Quil A, TiterMax, or IFA. Anti-PvMSP-3α in the sera of immunized mice was analyzed by ELISA 2 weeks after the first (**A**), second (**B**), and third (**C**) immunizing dose. Results are expressed as mean IgG antibody titers (log_10_) ± SEM and were compared by one-way ANOVA followed by Tukey’s test for multiple comparisons. Significant differences are noted on the graph: *p<0.05; **p<0.01; ***p<0.001. Non-significant (n.s.) differences are indicated (*p*>0.05). Data representative of 2 independent experiments.

**Figure 5:**
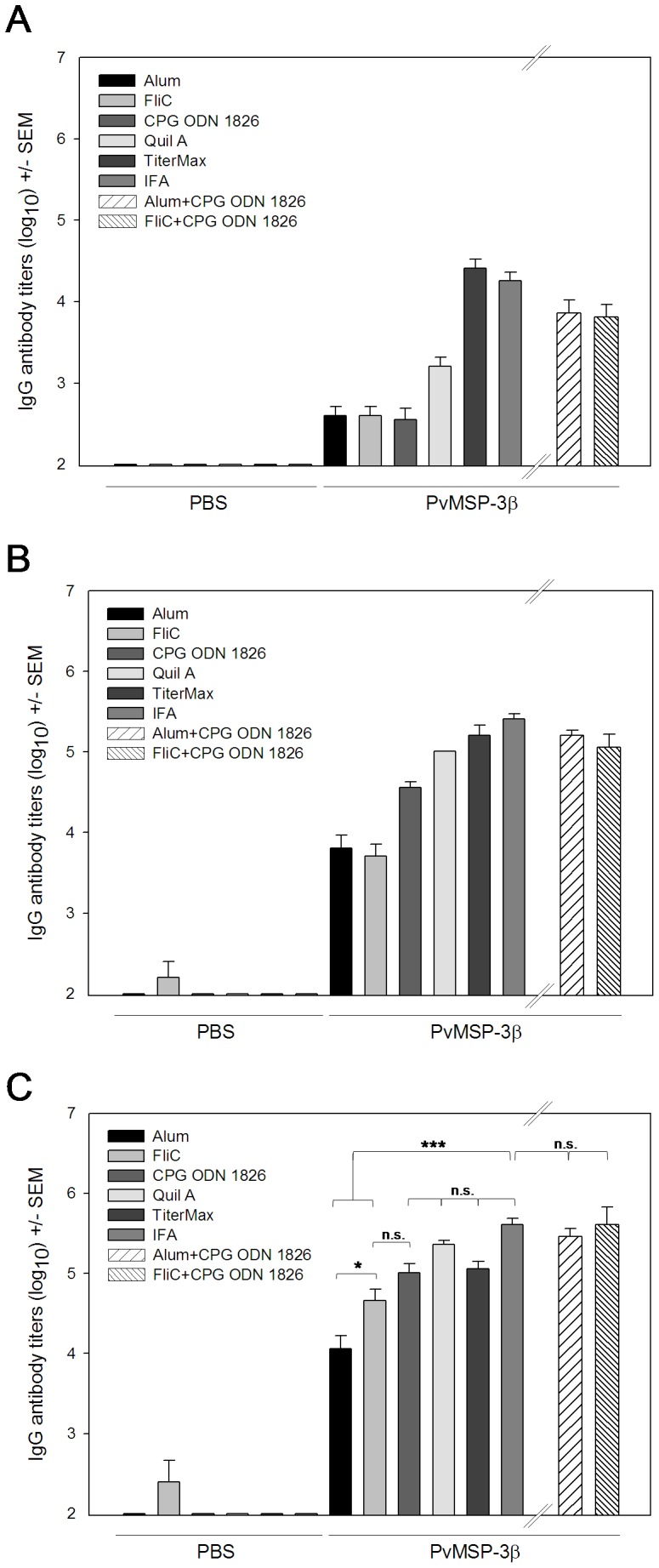
IgG anti-PvMSP-3β and IgG subclass profiles in mice immunized in the presence of adjuvant. Groups of 6 females BALB/c were immunized 3 times (s.c.) with 10 µg of protein in the presence of the following adjuvant formulations: Alum, FliC, CpG ODN 1826, Quil A, TiterMax, or IFA. The adjuvants Alum, FliC, and CpG ODN 1826 were also tested in combination (Alum plus CpG ODN 1826 and FliC+CpG ODN 1826). Anti-PvMSP-3β in the sera of immunized mice was analyzed by ELISA 2 weeks after the first (**A**), second (**B**), and third (**C**) doses. Results are expressed as mean IgG antibody titers (log_10_) ± SEM and were compared by one-way ANOVA followed by Tukey’s test for multiple comparisons. Significant differences are noted on the graph: *p<0.05; **p<0.01; ***p<0.001. Non-significant (n.s.) differences are indicated (*p*>0.05). Data representative of 2 independent experiments.

High antibody titers are desirable for vaccine efficacy. Toward that goal, we attempted to identify adjuvants that could significantly improve specific antibody responses. Our main goal was to obtain, if possible, titers similar to those elicited by immunization in the presence of Freund’s adjuvant. To test different adjuvant formulations, we selected 2 recombinant proteins, PvMSP-3α and full-length PvMSP-3β (FP-3). We used adjuvant formulations containing Alum, Quil A, TiterMax, IFA, and the TLR-5 or -9 agonists (FliC or CPG ODN 1826, respectively). Both recombinant proteins were highly immunogenic in BALB/c mice when administered in the presence of adjuvant (Figures 4 and 5). At the end of the immunization schedule, mice immunized with PvMSP-3α in Quil A, TiterMax, or IFA had significantly higher antibody titers than mice immunized with other adjuvant formulations (Figure 4C, *p*<0.001 in all cases). No statistically significant differences in antibody titers were detected in mice immunized with PvMSP-3α in Quil A, TiterMax, or IFA (*p>*0.05). However, mice immunized with PvMSP-3α formulated in CPG-ODN 1826 also had high antibody titers in comparison to FliC (*p<*0.05) or Alum (*p*<0.001). The mean antibody titers obtained with FliC and Alum did not significantly differ (*p*>0.05). Control mice immunized with adjuvants only did not present specific antibodies to PvMSP-3α throughout the experiment. The only exception was FliC-immunized mice, which had a low antibody immune response to PvMSP-3β (Figure 5). In this experiment, we observed that after the third dose, animals immunized with PvMSP-3β in CPG ODN 1826, Quil A, or TiterMax presented antibody titers similar to those of mice immunized with antigen in IFA (P>0.05) (Figure 5C). However, when administered with Alum or FliC antibody, titers to PvMSP-3β were significantly lower (*p*<0.001). FliC proved to be the most efficient adjuvant, generating antibody titers significantly higher than Alum (*p*<0.05). Interestingly, when CPG ODN 1826 was administered with Alum or FliC, it improved their activity, producing antibody titers as high as the IFA-immunized group (Figure 5C).

**Figure 6:**
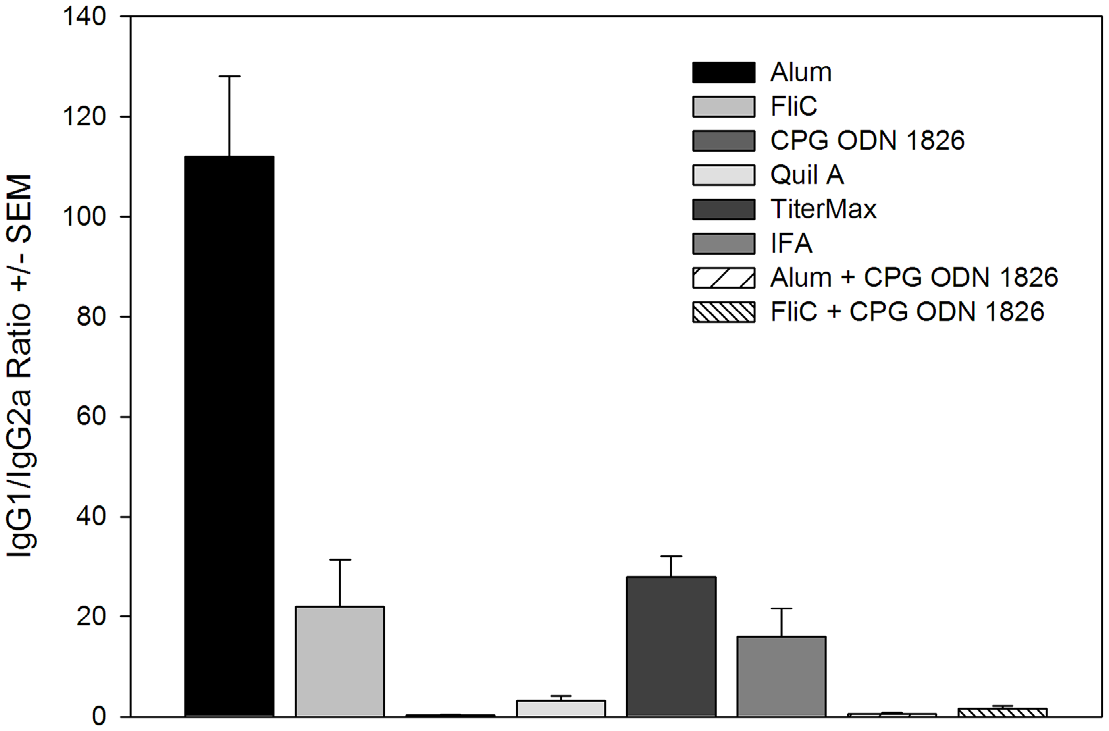
Serum IgG isotype responses in mice after immunization with PvMSP-3β in the presence of adjuvant. BALB/c mice were immunized with recombinant PvMSP-3β in the presence of adjuvant as described in Figure 5. PvMSP-3β-specific IgG1 and IgG2a antibody titers in the sera of immunized mice were analyzed by ELISA 2 weeks after the third dose. Results are the means of IgG1/IgG2a ± SEM for 6 mice per group. All groups were compared by one-way ANOVA and Tukey’s test for multiple comparisons.

The Th bias of the immune response was analyzed by determination of IgG subclasses in immunized by BALB/c mice. As shown in Figure 6, high levels of IgG1 were observed in mice immunized with Alum, in comparison to other groups, indicating Th2 polarization (IgG1/IgG2a ratio: 112). However, co-administration of Alum with CPG ODN 1826 greatly reduced the IgG1/IgG2a ratio in comparison to the adjuvant alone (Alum plus CPG ODN 1826∶112 vs. 0.36, *p*<0.05). Although co-administration of Alum plus FliC also modulated the Th1/Th2 response in comparison to FliC alone, this difference was not statistically significant (IgG1/IgG2a ratio: 22 vs. 1.63, *p*>0.05). A more balanced Th1/Th2 response was observed in mice immunized with PvMSP-3β in FliC, CPG-ODN 1826, Quil A, TiterMax, and IFA (IgG1/IgG2a ratio between 0.35 and 28).

In addition to IgG1 and IgG2a, in many cases, we have also detected the presence of IgG2b and IgG3. The results are summarized on Figure S1.

### Recognition of Native Protein in P. Vivax Parasites

**Figure 7:**
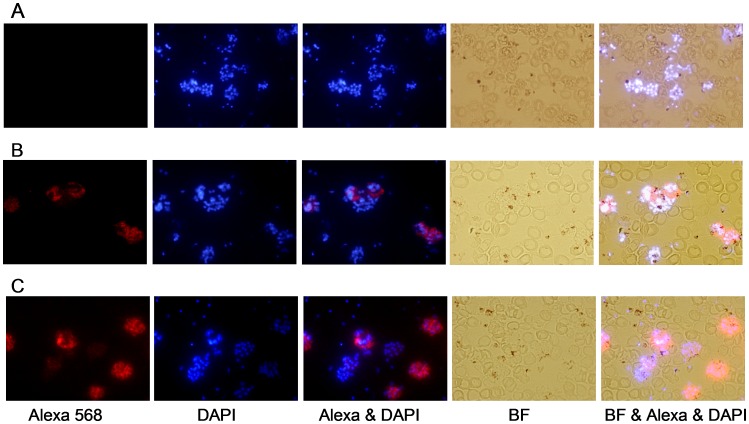
Immunofluorescence of *P. vivax* parasites and anti-MSP-3 antibodies. Immunofluorescence patterns in the sera of mice immunized with recombinant PvMSP-3α and PvMSP-3β (FP-3) on acetone-fixed *P. vivax*-infected erythrocytes (Pv-iE). The smears were incubated with pooled antisera (1∶100) from mice immunized with: (**A**) PBS emulsified in adjuvant, (**B**) PvMSP-3α, or (**C**) PvMSP-3β in Freund’s Adjuvant. Antibody binding was detected with secondary Alexa 568-labeled antibody (red) and nuclei were visualized by DAPI staining (blue). BF, bright field.

Pooled sera from mice immunized three times with PvMSP-3α and PvMSP-3β in Freund’s Adjuvant were tested for their ability to recognize native protein expressed by *P. vivax* merozoites. Both sera reacted with native protein exposed on the surface of *P. vivax* parasites isolated from an infected individual, but not with control sera. The IFA patterns obtained with these sera are shown in Figure 7, where the schizonts appeared as a “bunch of grapes” when stained [28]. It is important to note that some schizonts stain, but others do not. This fact could be explained by the presence in infected of patients of multiple clones of *P. vivax* parasites expressing multiple alleles of PvMSP-3α and PvMSP-3β.

## Discussion

We evaluated the immunogenic properties of four recombinant proteins representing MSP-3α and MSP-3β of *P. vivax*; one representing the C-terminal region of PvMSP-3α and three representing different regions of PvMSP-3β. Initially, these recombinant proteins were compared for their ability to bind IgG antibodies in the serum of individuals exposed to *P. vivax* malaria. We demonstrated that the frequencies of individuals with IgG antibodies to PvMSP-3α and at least one of the three recombinant proteins representing PvMSP-3β were relatively high (68.2% and 79.1%, respectively). For PvMSP-3α, these findings confirm two recent studies performed in distinct endemic areas of the Brazilian Amazon, where 78% [19] and 58.4% [20] of individuals presented IgG antibodies to this protein.

Previous studies evaluated the polymorphism in the C-terminal region of PvMSP-3α and found the region is highly conserved among natural isolates [35]. This fact may account for the recognition by IgG antibodies from a relatively high percentage of individuals. In contrast to the C-terminal region of PvMSP-3α, a variable degree of polymorphism has been reported for the gene encoding PvMSP-3β [36]. Despite the reported sequence diversity of PvMSP-3β, we found a significant percentage of individuals recognized PvMSP-3β recombinant proteins FP-2 and FP-3 (>60.0%). Nevertheless, we detected a lower frequency (26.3%) of responders to PvMSP-3β recombinant protein FP-1, suggesting most of the antibody responses were directed to the second moiety of the protein. The PvMSP-3α and PvMSP-3β polymorphic frequency in the studied areas is unknown. A recent study used PCR-RFLP to characterize the diversity of MSP-3α in 60 *P. vivax* isolates from four geographic regions of the Brazilian Amazon. The results revealed a high diversity where three different fragment sizes were found [37].

The cause for differential recognition by human antibodies of the N- and C-terminal regions of PvMSP-3β (FP-2 and FP-3) does not seem to be related to the absence of proper folding. In a previous study involving circular dichroism experiments, we demonstrated FP-1 was better structured than FP-2, which was highly recognized by IgG antibodies [31].

The comparison of human antibody reactivities to different antigens revealed major correlations. Significant correlations were observed between FP-2 and FP-3 of PvMSP-3β, possibly because they share a number of common B and T cell epitopes. However, the lack of correlation in most cases reflects differential genetic control by human HLA molecules. This hypothesis is being tested.

To investigate the immunogenic properties of PvMSP-3α and PvMSP-3β as vaccine candidates, we tested their immunogenicity in the presence or absence of different adjuvant systems in pre-clinical vaccinations of mice. In the absence of adjuvant, some of PvMSP-3β recombinant proteins elicited a specific TLR4-independent antibody response. This observation may explain how these molecules are immunogenic during natural human infection. In contrast, PvMSP-3α did not induce antibody immune responses, indicating the presence of other molecules in the parasite providing the adjuvant signal.

Previous studies have demonstrated that a major challenge in the development of subunit vaccines for malaria is the identification of a safe and potent adjuvant capable of inducing immune responses high antibody titers [38]. Antibody titers were very high in animals vaccinated with the C-terminus of PvMSP-3α or different regions of PvMSP-3β emulsified in IFA; thus, these recombinant proteins can be highly immunogenic. Our results indicate PvMSP-3α or a protein representing the majority of the PvMSP-3β sequence (FP-3) were immunogenic when administered in adjuvants other than IFA. The immunogenicity of PvMSP-3α was greater when administered in Quil A, a saponin derived from the bark of a Chilean tree, *Quillaja saponaria*, than in Alum, FliC, or CpG ODN 1826 and was similar to IFA. In addition, the antigen in TiterMax generated antibody titers similar to that obtained in IFA. PvMSP-3β yielded high antibody titers in all tested adjuvants, although Alum and FliC failed to perform at the level of IFA; however, TLR-9 agonist CPG ODN 1826 improved their adjuvant activity. It is of interest to note that adjuvants such as Alum, FliC, TiterMax, and IFA tend to induce Th2 with high IgG1/IgG2a ratios, whereas CPG ODN 1826 and Quil A show a clear modulation of the IgG subclass response pattern to a more balanced Th1/Th2 response.

The cellular response to CpG DNA is mediated by TLR9, followed by induction of pro-inflammatory cytokines (*e.g.* IL-12, TNF-α, and IFN-γ), and producing a strong Th1 response [39]. We observed a response pattern favoring Th1 in all formulations containing CPG ODN (CPG ODN 1826 alone, Alum+CPG ODN 1826, and FliC+CPG ODN 1826). Clinical trials evaluating the adjuvant activity of CpG ODN with vaccines designed to prevent malaria have been reported [40]–[43]. Co-administration of CpG with AMA-1 [40] or MSP1_42_ of *P. falciparum* [43] increased the geometric media of antibodies by 5.5 or 8-fold, respectively, when compared to each protein alone.

The relevance of antibodies against PvMSP-3α or β in host protection remains untested. Evidence in favor of a protective role for anti-*P. vivax* MSP-3 was obtained by clinical trials performed with *P. falciparum* MSP3.1 [3]. The functional role(s) for the parasite and in the context of host immune responses remain to be determined for other members of the MSP3 family in each of these species [32]. Such investigations as a whole should help guide decisions for the development of malaria vaccines based on these or alternative proteins, which could prove to be valuable in areas of the world afflicted with both *P. falciparum* and *P. vivax*.

## Supporting Information

Figure S1 **Serum IgG isotype responses in mice after immunization with PvMSP-3 in the presence of adjuvant.** BALB/c mice were immunized with the recombinant proteins PvMSP-3α (A) or PvMSP-3β FP-3 (B) in the presence of adjuvant as described in Figures 4 and 5. IgG1, IgG2a, IgG2b and IgG3 antibody titers in the sera of immunized mice were analyzed by ELISA 2 weeks after the third dose. Results are expressed as mean IgG antibody titers (log_10_) ± SEM for 6 mice per group.
(TIF)
