# Supplementary figures and images for: Antigenicity and Immunogenicity of Plasmodium vivax Merozoite Surface Protein-3
Source: PLoS One. 2013 Feb 14;8(2):e56061. doi: 10.1371/journal.pone.0056061 (PMC3573074; doi:10.1371/journal.pone.0056061)

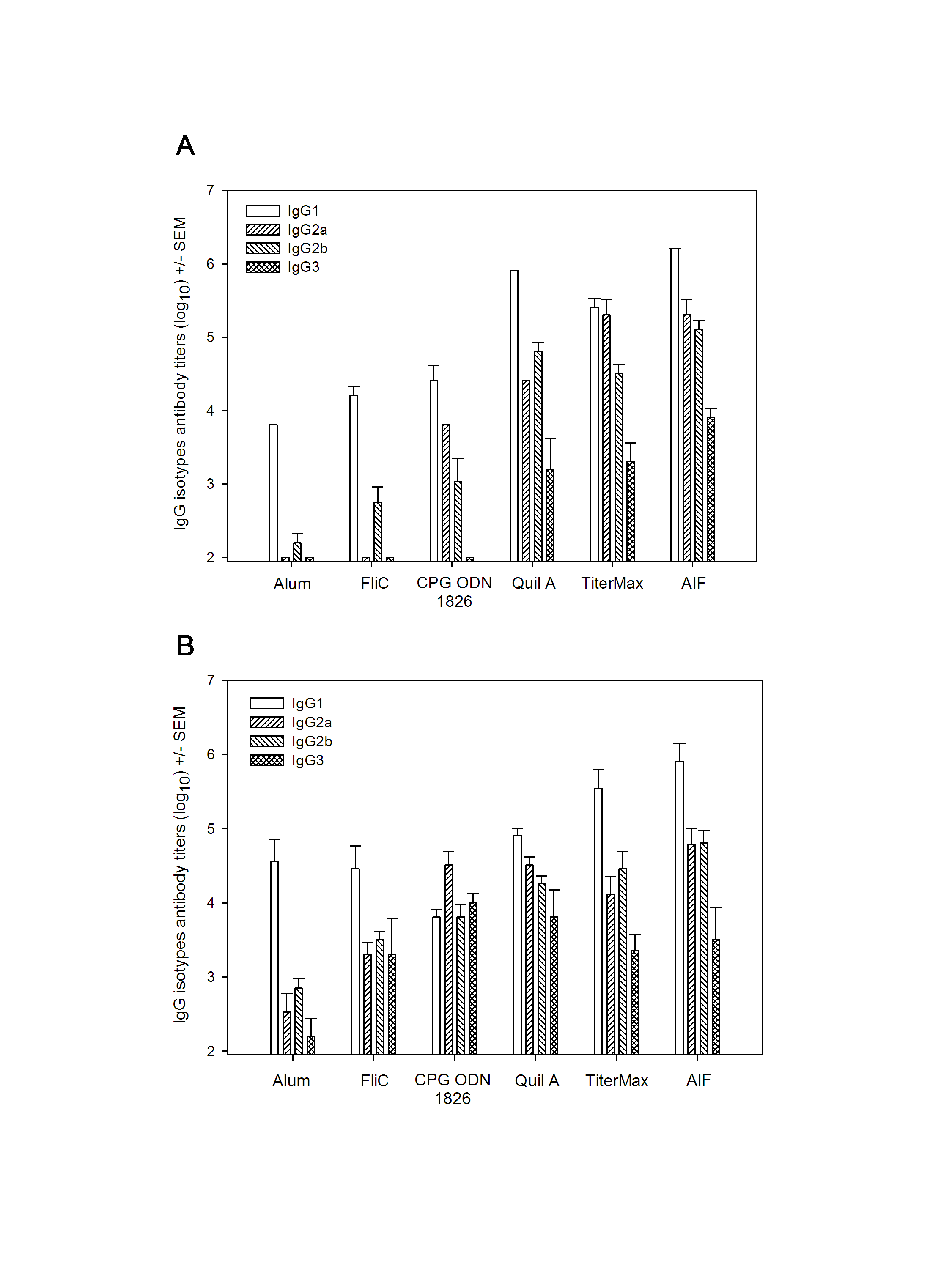

Supplement: Figure S1 — Serum IgG isotype responses in mice after immunization with PvMSP-3 in the presence of adjuvant. BALB/c mice were immunized with the recombinant proteins PvMSP-3α (A) or PvMSP-3β FP-3 (B) in the presence of adjuvant as described in Figures 4 and 5. IgG1, IgG2a, IgG2b and IgG3 antibody titers in the sera of immunized mice were analyzed by ELISA 2 weeks after the third dose. Results are expressed as mean IgG antibody titers (log10) ± SEM for 6 mice per group. (TIF) [file pone.0056061.s001.tif]
